# 4,6-Dimethyl­pyrimidin-2-amine

**DOI:** 10.1107/S1600536812049458

**Published:** 2012-12-08

**Authors:** Wei-Wei Fu, Yang Liu, Geng Huang, Xiao-Ming Zhu

**Affiliations:** a Key Laboratory of Functional Organometallic Materials of General Colleges and Universities in Hunan Province, Department of Chemistry and Materials Science, Hengyang Normal University, Hengyang 421008, People’s Republic of China

## Abstract

The asymmetric unit of the title compound, C_6_H_9_N_3_, contains three crystallographically independent mol­ecules of similar geometry. All of the mol­ecules are almost planar, with r.m.s. deviations of 0.003, 0.016 and 0.005 Å. In the crystal, the mol­ecules are linked by N—H⋯N hydrogen bonds into zigzag ribbons parallel to the *c* axis, generating rings of *R*_2_^2^(8) graph-set motif.

## Related literature

For background to sulfonyl­urea herbicides, see: Deng (2003 ▶). For the properties and crystal structures of metal complexes of the title compound, see: Sun *et al.* (2010 ▶); Yang (2009 ▶). For the structure of a hydrate form of the title compound, see: Lin *et al.* (2008 ▶). For the synthesis, see: Fan *et al.* (2000 ▶); Yao & Qu (1997 ▶).
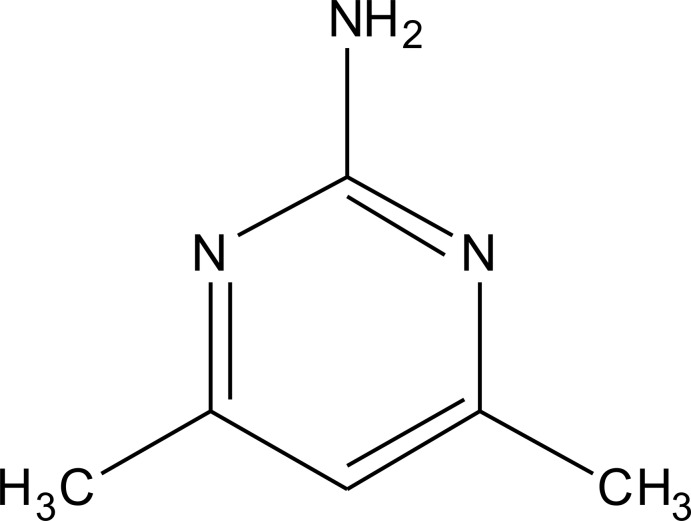


## Experimental

### 

#### Crystal data

C_6_H_9_N_3_
*M* *_r_* = 123.16
Monoclinic, 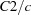

*a* = 11.519 (7) Å
*b* = 11.021 (6) Å
*c* = 32.386 (18) Å
β = 91.112 (10)°
*V* = 4111 (4) Å^3^
*Z* = 24
Mo *K*α radiation
μ = 0.08 mm^−1^
*T* = 296 K
0.26 × 0.18 × 0.17 mm

#### Data collection

Bruker APEXII CCD diffractometer
Absorption correction: multi-scan (*SADABS*; Bruker, 2001 ▶) *T*_min_ = 0.980, *T*_max_ = 0.987
11005 measured reflections
4020 independent reflections
2252 reflections with *I* > 2σ(*I*)
*R*_int_ = 0.032

#### Refinement

*R*[*F* ^2^ > 2σ(*F* ^2^)] = 0.062
*wR*(*F* ^2^) = 0.175
*S* = 1.01
4020 reflections
245 parameters
H-atom parameters constrained
Δρ_max_ = 1.08 e Å^−3^
Δρ_min_ = −0.16 e Å^−3^

### 

Data collection: *APEX2* (Bruker, 2007 ▶); cell refinement: *SAINT* (Bruker, 2007 ▶); data reduction: *SAINT*; program(s) used to solve structure: *SHELXS97* (Sheldrick, 2008 ▶); program(s) used to refine structure: *SHELXL97* (Sheldrick, 2008 ▶); molecular graphics: *SHELXTL* (Sheldrick, 2008 ▶) and *DIAMOND* (Brandenburg, 2008 ▶); software used to prepare material for publication: *SHELXTL* and *publCIF* (Westrip, 2010 ▶).

## Supplementary Material

Crystal structure: contains datablock(s) I, global. DOI: 10.1107/S1600536812049458/rz5030sup1.cif

Structure factors: contains datablock(s) I. DOI: 10.1107/S1600536812049458/rz5030Isup2.hkl

Supplementary material file. DOI: 10.1107/S1600536812049458/rz5030Isup3.cml

Additional supplementary materials:  crystallographic information; 3D view; checkCIF report

## Figures and Tables

**Table 1:** Hydrogen-bond geometry (Å, °)

*D*—H⋯*A*	*D*—H	H⋯*A*	*D*⋯*A*	*D*—H⋯*A*
N1—H1*A*⋯N2^i^	0.86	2.45	3.304 (4)	175
N1—H1*B*⋯N8^ii^	0.86	2.52	3.376 (4)	173
N4—H4*A*⋯N9^iii^	0.86	2.21	3.050 (3)	167
N4—H4*B*⋯N6^iv^	0.86	2.45	3.243 (4)	154
N7—H7*A*⋯N5^v^	0.86	2.57	3.421 (4)	173
N7—H7*B*⋯N3^vi^	0.86	2.36	3.219 (3)	176

Symmetry codes: (i) 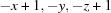; (ii) 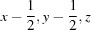; (iii) 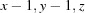; (iv) 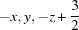; (v) 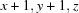; (vi) 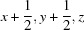.

**Fig. 1.**
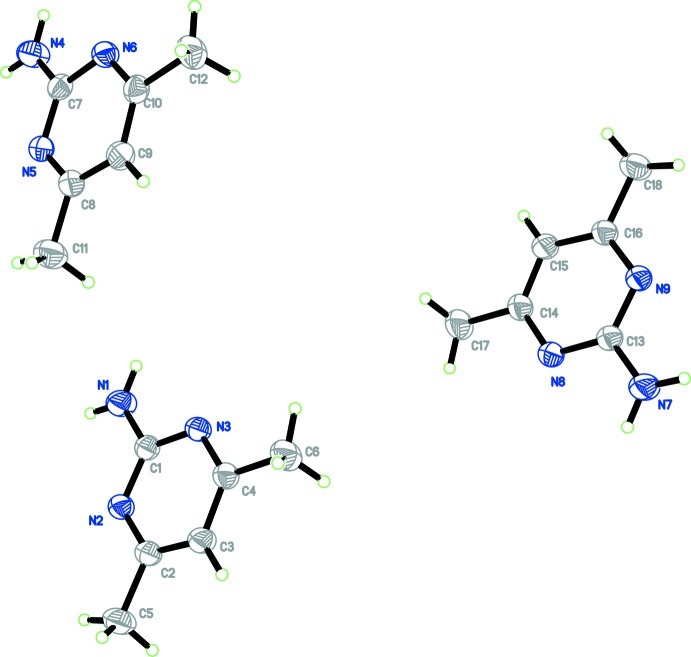
ORTEP plot of the asymmetric unit of the title compound with displacement ellipsoids drawn at the 30% probability level.

**Fig. 2.**
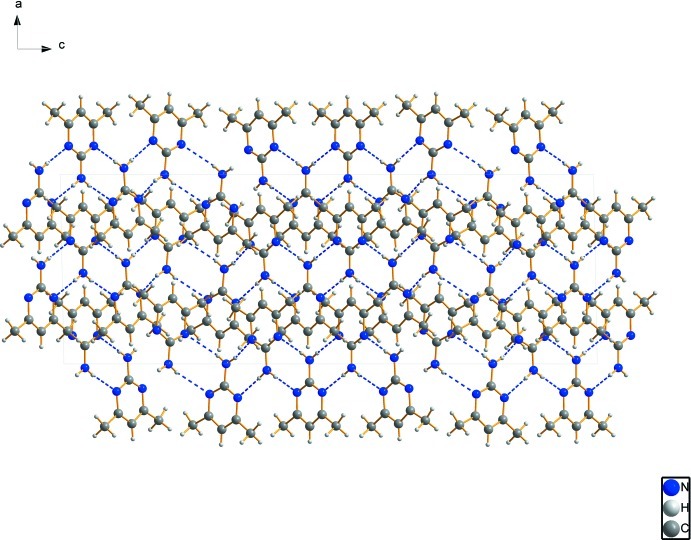
Packing diagram of the title compound viewed along the b axis. Hydrogen bonds are drawn as dashed lines.

## supplementary crystallographic information

### Comment

4,6-Dimethylpyrimidin-2-amine is an important intermediate especially for synthesis of sulfonylurea herbicides (Deng, 2003). It has also been used as an organic ligand in the fluorescence research on Ag(I) coordination complexes (Sun *et al.*, 2010; Yang, 2009). As a continuation of our efforts aimed to the synthesis of new ligands based on this organic compound, the title compound has been unexpectedly obtained and its crystal structure is reported herein.

The asymmetric unit of the title compound consists of three crystallographically independent molecules of similar geometry (Fig. 1). All molecules are substantially planar, the r.m.s. deviations being 0.003, 0.016 and 0.005 Å for N1–N3/C1–C6, N4–N6/C7–C12 and N7–N9/C13–C18 respectively. In the crystal structure (Fig. 2), molecules are connected by classical intermolecular N—H···N hydrogen bonds (Table 1) to form zigzag ribbons parallel to the *c* axis generating rings of *R*^2^_2_(8) graph set motif. The structure of a hydrate form of the title compound was reported recently (Lin *et al.*, 2008).

### Experimental

In an attempt to synthesize 1,3-bis(4,6-dimethylpyrimidin-2-yl)thiourea according to a literature method (Fan *et al.*, 2000), 4,6-dimethylpyrimidin-2-amine (Yao & Qu, 1997) was used as material as a replacement for pyridin-2-amine. Sodium hydroxide (0.25 g, 6.25 mmol) was dissolved in absolute ethanol (25 ml), then 4,6-dimethylpyrimidin-2-amine (6.15 g, 50 mmol) and carbon disulfide (2.25 g, 30 mmol) were added. After refluxing for 6 h, the mixture was cooled and filtered. The title compound was obtained as colourless crystals suitable for X-ray diffraction instead of the expected thiourea derivative. All reagents and solvents were commercially available and used without further purification.

### Refinement

All non-H atoms were refined anisotropically. Hydrogen atoms were positioned geometrically and treated as riding atoms, with C—H = 0.90–1.00 Å, N—H = 0.88–0.91 Å and with with *U*_iso_(H) = 1.2*U*_eq_(C, N) or 1.5*U*_eq_(C) for methyl H atoms. The relatively high residual peak of 1.08 e/Å^3^ is located on a twofold axis.

### Crystal data

**Table:**

C_6_H_9_N_3_	*F*(000) = 1584
*M**_r_* = 123.16	*D*_x_ = 1.194 Mg m^−^^3^
Monoclinic, *C*2/*c*	Mo *K*α radiation, λ = 0.71073 Å
Hall symbol: -C 2yc	Cell parameters from 1760 reflections
*a* = 11.519 (7) Å	θ = 2.6–23.0°
*b* = 11.021 (6) Å	µ = 0.08 mm^−^^1^
*c* = 32.386 (18) Å	*T* = 296 K
β = 91.112 (10)°	Block, colourless
*V* = 4111 (4) Å^3^	0.26 × 0.18 × 0.17 mm
*Z* = 24	

### Data collection

**Table:**

Bruker APEXII CCD diffractometer	4020 independent reflections
Radiation source: fine-focus sealed tube	2252 reflections with *I* > 2σ(*I*)
Graphite monochromator	*R*_int_ = 0.032
φ and ω scans	θ_max_ = 26.0°, θ_min_ = 2.6°
Absorption correction: multi-scan (*SADABS*; Bruker, 2001)	*h* = −14→14
*T*_min_ = 0.980, *T*_max_ = 0.987	*k* = −13→6
11005 measured reflections	*l* = −39→39

### Refinement

**Table:**

Refinement on *F*^2^	Secondary atom site location: difference Fourier map
Least-squares matrix: full	Hydrogen site location: inferred from neighbouring sites
*R*[*F*^2^ > 2σ(*F*^2^)] = 0.062	H-atom parameters constrained
*wR*(*F*^2^) = 0.175	*w* = 1/[σ^2^(*F*_o_^2^) + (0.0567*P*)^2^ + 5.4094*P*] where *P* = (*F*_o_^2^ + 2*F*_c_^2^)/3
*S* = 1.01	(Δ/σ)_max_ < 0.001
4020 reflections	Δρ_max_ = 1.08 e Å^−^^3^
245 parameters	Δρ_min_ = −0.16 e Å^−^^3^
0 restraints	Extinction correction: *SHELXL97* (Sheldrick, 2008), Fc^*^=kFc[1+0.001xFc^2^λ^3^/sin(2θ)]^-1/4^
Primary atom site location: structure-invariant direct methods	Extinction coefficient: 0.0011 (2)

### Special details

**Table:**

Geometry. All e.s.d.'s (except the e.s.d. in the dihedral angle between two l.s. planes) are estimated using the full covariance matrix. The cell e.s.d.'s are taken into account individually in the estimation of e.s.d.'s in distances, angles and torsion angles; correlations between e.s.d.'s in cell parameters are only used when they are defined by crystal symmetry. An approximate (isotropic) treatment of cell e.s.d.'s is used for estimating e.s.d.'s involving l.s. planes.
Refinement. Refinement of *F*^2^ against ALL reflections. The weighted *R*-factor *wR* and goodness of fit *S* are based on *F*^2^, conventional *R*-factors *R* are based on *F*, with *F* set to zero for negative *F*^2^. The threshold expression of *F*^2^ > σ(*F*^2^) is used only for calculating *R*-factors(gt) *etc*. and is not relevant to the choice of reflections for refinement. *R*-factors based on *F*^2^ are statistically about twice as large as those based on *F*, and *R*- factors based on ALL data will be even larger.

### Fractional atomic coordinates and isotropic or equivalent isotropic displacement parameters (Å^2^)

**Table:**

	*x*	*y*	*z*	*U*_iso_*/*U*_eq_	
N1	0.47955 (19)	0.1483 (2)	0.53927 (8)	0.0677 (7)	
H1A	0.4423	0.0961	0.5244	0.081*	
H1B	0.4422	0.2003	0.5537	0.081*	
N2	0.64988 (18)	0.0645 (2)	0.51683 (7)	0.0514 (6)	
N3	0.65035 (18)	0.2326 (2)	0.56391 (6)	0.0497 (6)	
N4	−0.0008 (2)	0.2232 (2)	0.69589 (8)	0.0702 (8)	
H4A	−0.0320	0.1741	0.6783	0.084*	
H4B	−0.0438	0.2682	0.7110	0.084*	
N5	0.17824 (19)	0.1573 (2)	0.67544 (7)	0.0513 (6)	
N6	0.15897 (19)	0.3101 (2)	0.72784 (7)	0.0528 (6)	
N7	1.03321 (19)	0.9393 (2)	0.61966 (8)	0.0705 (8)	
H7A	1.0747	0.9889	0.6342	0.085*	
H7B	1.0662	0.8868	0.6042	0.085*	
N8	0.85527 (19)	0.8637 (2)	0.59788 (7)	0.0522 (6)	
N9	0.87060 (18)	1.0286 (2)	0.64615 (7)	0.0507 (6)	
C1	0.5969 (2)	0.1484 (2)	0.54006 (8)	0.0481 (6)	
C2	0.7663 (2)	0.0667 (2)	0.51776 (8)	0.0533 (7)	
C3	0.8282 (2)	0.1495 (3)	0.54127 (9)	0.0572 (7)	
H3A	0.9090	0.1498	0.5416	0.069*	
C4	0.7666 (2)	0.2320 (2)	0.56421 (8)	0.0515 (7)	
C5	0.8265 (3)	−0.0260 (3)	0.49198 (11)	0.0766 (10)	
H5A	0.7697	−0.0748	0.4776	0.115*	
H5B	0.8739	−0.0769	0.5095	0.115*	
H5C	0.8745	0.0142	0.4723	0.115*	
C6	0.8267 (3)	0.3251 (3)	0.59082 (11)	0.0743 (9)	
H6A	0.7698	0.3739	0.6043	0.111*	
H6B	0.8741	0.3759	0.5740	0.111*	
H6C	0.8747	0.2850	0.6112	0.111*	
C7	0.1161 (2)	0.2298 (2)	0.69984 (8)	0.0487 (6)	
C8	0.2939 (2)	0.1687 (2)	0.67901 (9)	0.0547 (7)	
C9	0.3449 (2)	0.2505 (3)	0.70623 (9)	0.0597 (8)	
H9A	0.4252	0.2582	0.7081	0.072*	
C10	0.2743 (2)	0.3200 (2)	0.73041 (8)	0.0520 (7)	
C11	0.3651 (3)	0.0882 (3)	0.65230 (12)	0.0846 (11)	
H11A	0.3146	0.0383	0.6356	0.127*	
H11B	0.4139	0.0375	0.6694	0.127*	
H11C	0.4125	0.1370	0.6348	0.127*	
C12	0.3216 (3)	0.4120 (3)	0.76051 (10)	0.0744 (9)	
H12A	0.2585	0.4510	0.7742	0.112*	
H12B	0.3659	0.4716	0.7460	0.112*	
H12C	0.3707	0.3722	0.7806	0.112*	
C13	0.9157 (2)	0.9440 (2)	0.62120 (8)	0.0490 (6)	
C14	0.7390 (2)	0.8696 (3)	0.60022 (9)	0.0546 (7)	
C15	0.6851 (2)	0.9530 (3)	0.62522 (9)	0.0582 (7)	
H15A	0.6046	0.9561	0.6267	0.070*	
C16	0.7545 (2)	1.0319 (2)	0.64800 (8)	0.0515 (7)	
C17	0.6703 (3)	0.7818 (3)	0.57420 (11)	0.0779 (10)	
H17A	0.7223	0.7314	0.5590	0.117*	
H17B	0.6208	0.8257	0.5553	0.117*	
H17C	0.6237	0.7319	0.5917	0.117*	
C18	0.7026 (3)	1.1255 (3)	0.67570 (10)	0.0759 (10)	
H18A	0.7635	1.1713	0.6890	0.114*	
H18B	0.6569	1.0860	0.6962	0.114*	
H18C	0.6540	1.1790	0.6596	0.114*	

### Atomic displacement parameters (Å^2^)

**Table:**

	*U*^11^	*U*^22^	*U*^33^	*U*^12^	*U*^13^	*U*^23^
N1	0.0420 (13)	0.0787 (17)	0.0825 (18)	−0.0024 (12)	0.0011 (12)	−0.0227 (14)
N2	0.0469 (13)	0.0528 (13)	0.0547 (13)	−0.0021 (11)	0.0025 (10)	−0.0097 (11)
N3	0.0450 (13)	0.0505 (13)	0.0538 (13)	0.0006 (10)	0.0035 (10)	−0.0100 (11)
N4	0.0462 (14)	0.0892 (19)	0.0751 (17)	−0.0057 (13)	0.0016 (12)	−0.0298 (15)
N5	0.0496 (14)	0.0496 (13)	0.0548 (14)	−0.0051 (11)	0.0059 (10)	−0.0054 (11)
N6	0.0499 (14)	0.0566 (14)	0.0518 (13)	−0.0050 (11)	0.0025 (10)	−0.0091 (11)
N7	0.0424 (13)	0.0830 (18)	0.0862 (18)	0.0016 (13)	0.0053 (12)	−0.0293 (15)
N8	0.0469 (13)	0.0525 (13)	0.0573 (14)	0.0002 (11)	0.0033 (10)	−0.0094 (11)
N9	0.0424 (12)	0.0534 (13)	0.0562 (13)	−0.0001 (10)	0.0025 (10)	−0.0093 (11)
C1	0.0442 (15)	0.0495 (15)	0.0504 (15)	−0.0010 (12)	0.0005 (12)	−0.0031 (13)
C2	0.0503 (16)	0.0511 (16)	0.0586 (17)	0.0019 (13)	0.0052 (13)	−0.0093 (13)
C3	0.0406 (15)	0.0619 (17)	0.0690 (18)	−0.0018 (13)	0.0024 (13)	−0.0153 (15)
C4	0.0465 (15)	0.0505 (15)	0.0575 (16)	−0.0027 (13)	−0.0001 (12)	−0.0095 (13)
C5	0.066 (2)	0.077 (2)	0.087 (2)	0.0056 (17)	0.0113 (17)	−0.0316 (19)
C6	0.0566 (19)	0.074 (2)	0.092 (2)	−0.0037 (16)	−0.0060 (16)	−0.0336 (19)
C7	0.0457 (15)	0.0527 (16)	0.0479 (15)	−0.0060 (13)	0.0017 (12)	0.0012 (13)
C8	0.0515 (16)	0.0489 (16)	0.0639 (18)	−0.0029 (13)	0.0077 (13)	−0.0053 (14)
C9	0.0430 (15)	0.0604 (18)	0.076 (2)	−0.0040 (14)	0.0040 (14)	−0.0075 (16)
C10	0.0478 (16)	0.0527 (16)	0.0555 (16)	−0.0090 (13)	−0.0006 (12)	−0.0022 (13)
C11	0.061 (2)	0.082 (2)	0.111 (3)	−0.0008 (18)	0.0156 (19)	−0.033 (2)
C12	0.068 (2)	0.073 (2)	0.081 (2)	−0.0123 (17)	−0.0058 (17)	−0.0222 (18)
C13	0.0434 (15)	0.0514 (15)	0.0522 (15)	0.0018 (12)	0.0034 (12)	−0.0037 (13)
C14	0.0506 (17)	0.0541 (16)	0.0591 (17)	−0.0033 (13)	−0.0001 (13)	−0.0081 (14)
C15	0.0413 (15)	0.0625 (18)	0.0709 (19)	0.0003 (14)	0.0044 (13)	−0.0128 (15)
C16	0.0449 (15)	0.0538 (16)	0.0559 (16)	0.0023 (13)	0.0027 (12)	−0.0085 (13)
C17	0.062 (2)	0.077 (2)	0.095 (2)	−0.0103 (17)	−0.0020 (17)	−0.032 (2)
C18	0.0580 (19)	0.083 (2)	0.087 (2)	0.0058 (17)	0.0102 (16)	−0.0310 (19)

### Geometric parameters (Å, º)

**Table:**

N1—C1	1.351 (3)	C5—H5B	0.9600
N1—H1A	0.8600	C5—H5C	0.9600
N1—H1B	0.8600	C6—H6A	0.9600
N2—C2	1.341 (3)	C6—H6B	0.9600
N2—C1	1.346 (3)	C6—H6C	0.9600
N3—C4	1.339 (3)	C8—C9	1.383 (4)
N3—C1	1.349 (3)	C8—C11	1.496 (4)
N4—C7	1.351 (3)	C9—C10	1.373 (4)
N4—H4A	0.8600	C9—H9A	0.9300
N4—H4B	0.8600	C10—C12	1.502 (4)
N5—C8	1.341 (3)	C11—H11A	0.9600
N5—C7	1.341 (3)	C11—H11B	0.9600
N6—C10	1.334 (3)	C11—H11C	0.9600
N6—C7	1.354 (3)	C12—H12A	0.9600
N7—C13	1.357 (3)	C12—H12B	0.9600
N7—H7A	0.8600	C12—H12C	0.9600
N7—H7B	0.8600	C14—C15	1.380 (4)
N8—C14	1.345 (3)	C14—C17	1.499 (4)
N8—C13	1.348 (3)	C15—C16	1.384 (4)
N9—C16	1.340 (3)	C15—H15A	0.9300
N9—C13	1.344 (3)	C16—C18	1.499 (4)
C2—C3	1.379 (4)	C17—H17A	0.9600
C2—C5	1.499 (4)	C17—H17B	0.9600
C3—C4	1.380 (4)	C17—H17C	0.9600
C3—H3A	0.9300	C18—H18A	0.9600
C4—C6	1.500 (4)	C18—H18B	0.9600
C5—H5A	0.9600	C18—H18C	0.9600
			
C1—N1—H1A	120.0	C9—C8—C11	121.6 (3)
C1—N1—H1B	120.0	C10—C9—C8	118.5 (3)
H1A—N1—H1B	120.0	C10—C9—H9A	120.7
C2—N2—C1	116.1 (2)	C8—C9—H9A	120.7
C4—N3—C1	116.5 (2)	N6—C10—C9	121.3 (3)
C7—N4—H4A	120.0	N6—C10—C12	116.4 (3)
C7—N4—H4B	120.0	C9—C10—C12	122.4 (3)
H4A—N4—H4B	120.0	C8—C11—H11A	109.5
C8—N5—C7	115.7 (2)	C8—C11—H11B	109.5
C10—N6—C7	116.5 (2)	H11A—C11—H11B	109.5
C13—N7—H7A	120.0	C8—C11—H11C	109.5
C13—N7—H7B	120.0	H11A—C11—H11C	109.5
H7A—N7—H7B	120.0	H11B—C11—H11C	109.5
C14—N8—C13	116.1 (2)	C10—C12—H12A	109.5
C16—N9—C13	116.3 (2)	C10—C12—H12B	109.5
N2—C1—N3	125.8 (2)	H12A—C12—H12B	109.5
N2—C1—N1	116.9 (2)	C10—C12—H12C	109.5
N3—C1—N1	117.2 (2)	H12A—C12—H12C	109.5
N2—C2—C3	122.1 (2)	H12B—C12—H12C	109.5
N2—C2—C5	116.7 (3)	N9—C13—N8	126.2 (2)
C3—C2—C5	121.2 (3)	N9—C13—N7	116.5 (2)
C2—C3—C4	117.8 (3)	N8—C13—N7	117.3 (2)
C2—C3—H3A	121.1	N8—C14—C15	121.7 (3)
C4—C3—H3A	121.1	N8—C14—C17	116.9 (3)
N3—C4—C3	121.6 (2)	C15—C14—C17	121.4 (3)
N3—C4—C6	116.8 (2)	C14—C15—C16	118.0 (3)
C3—C4—C6	121.5 (3)	C14—C15—H15A	121.0
C2—C5—H5A	109.5	C16—C15—H15A	121.0
C2—C5—H5B	109.5	N9—C16—C15	121.7 (2)
H5A—C5—H5B	109.5	N9—C16—C18	117.1 (2)
C2—C5—H5C	109.5	C15—C16—C18	121.2 (2)
H5A—C5—H5C	109.5	C14—C17—H17A	109.5
H5B—C5—H5C	109.5	C14—C17—H17B	109.5
C4—C6—H6A	109.5	H17A—C17—H17B	109.5
C4—C6—H6B	109.5	C14—C17—H17C	109.5
H6A—C6—H6B	109.5	H17A—C17—H17C	109.5
C4—C6—H6C	109.5	H17B—C17—H17C	109.5
H6A—C6—H6C	109.5	C16—C18—H18A	109.5
H6B—C6—H6C	109.5	C16—C18—H18B	109.5
N5—C7—N4	117.0 (2)	H18A—C18—H18B	109.5
N5—C7—N6	126.3 (2)	C16—C18—H18C	109.5
N4—C7—N6	116.6 (2)	H18A—C18—H18C	109.5
N5—C8—C9	121.7 (3)	H18B—C18—H18C	109.5
N5—C8—C11	116.7 (3)		
			
C2—N2—C1—N3	−0.3 (4)	N5—C8—C9—C10	0.8 (4)
C2—N2—C1—N1	179.5 (3)	C11—C8—C9—C10	−178.7 (3)
C4—N3—C1—N2	0.2 (4)	C7—N6—C10—C9	−1.0 (4)
C4—N3—C1—N1	−179.7 (3)	C7—N6—C10—C12	178.1 (2)
C1—N2—C2—C3	0.3 (4)	C8—C9—C10—N6	−0.3 (4)
C1—N2—C2—C5	−179.9 (3)	C8—C9—C10—C12	−179.3 (3)
N2—C2—C3—C4	−0.2 (4)	C16—N9—C13—N8	0.6 (4)
C5—C2—C3—C4	−179.9 (3)	C16—N9—C13—N7	−179.2 (3)
C1—N3—C4—C3	0.0 (4)	C14—N8—C13—N9	−0.2 (4)
C1—N3—C4—C6	−179.7 (3)	C14—N8—C13—N7	179.5 (3)
C2—C3—C4—N3	0.0 (4)	C13—N8—C14—C15	−0.2 (4)
C2—C3—C4—C6	179.7 (3)	C13—N8—C14—C17	179.5 (3)
C8—N5—C7—N4	177.8 (3)	N8—C14—C15—C16	0.3 (4)
C8—N5—C7—N6	−1.5 (4)	C17—C14—C15—C16	−179.4 (3)
C10—N6—C7—N5	2.0 (4)	C13—N9—C16—C15	−0.4 (4)
C10—N6—C7—N4	−177.3 (2)	C13—N9—C16—C18	179.9 (3)
C7—N5—C8—C9	0.0 (4)	C14—C15—C16—N9	0.1 (4)
C7—N5—C8—C11	179.6 (3)	C14—C15—C16—C18	179.7 (3)

### Hydrogen-bond geometry (Å, º)

**Table:**

*D*—H···*A*	*D*—H	H···*A*	*D*···*A*	*D*—H···*A*
N1—H1*A*···N2^i^	0.86	2.45	3.304 (4)	175
N1—H1*B*···N8^ii^	0.86	2.52	3.376 (4)	173
N4—H4*A*···N9^iii^	0.86	2.21	3.050 (3)	167
N4—H4*B*···N6^iv^	0.86	2.45	3.243 (4)	154
N7—H7*A*···N5^v^	0.86	2.57	3.421 (4)	173
N7—H7*B*···N3^vi^	0.86	2.36	3.219 (3)	176

Symmetry codes: (i) −*x*+1, −*y*, −*z*+1; (ii) *x*−1/2, *y*−1/2, *z*; (iii) *x*−1, *y*−1, *z*; (iv) −*x*, *y*, −*z*+3/2; (v) *x*+1, *y*+1, *z*; (vi) *x*+1/2, *y*+1/2, *z*.
